# I Like, I Cite? Do Facebook Likes Predict the Impact of Scientific Work?

**DOI:** 10.1371/journal.pone.0134389

**Published:** 2015-08-05

**Authors:** Stefanie Ringelhan, Jutta Wollersheim, Isabell M. Welpe

**Affiliations:** 1 Chair for Strategy and Organization, TUM School of Management, Technische Universität München, Munich, Bavaria, Germany; 2 Bavarian State Institute for Higher Education Research and Planning, Munich, Bavaria, Germany; Universidad de Las Palmas de Gran Canaria, SPAIN

## Abstract

Due to the increasing amount of scientific work and the typical delays in publication, promptly assessing the impact of scholarly work is a huge challenge. To meet this challenge, one solution may be to create and discover innovative indicators. The goal of this paper is to investigate whether Facebook likes for unpublished manuscripts that are uploaded to the Internet could be used as an early indicator of the future impact of the scientific work. To address our research question, we compared Facebook likes for manuscripts uploaded to the Harvard Business School website (Study 1) and the bioRxiv website (Study 2) with traditional impact indicators (journal article citations, Impact Factor, Immediacy Index) for those manuscripts that have been published as a journal article. Although based on our full sample of Study 1 (*N* = 170), Facebook likes do not predict traditional impact indicators, for manuscripts with one or more Facebook likes (*n* = 95), our results indicate that the more Facebook likes a manuscript receives, the more journal article citations the manuscript receives. In additional analyses (for which we categorized the manuscripts as psychological and non-psychological manuscripts), we found that the significant prediction of citations stems from the psychological and not the non-psychological manuscripts. In Study 2, we observed that Facebook likes (*N* = 270) and non-zero Facebook likes (*n* = 84) do not predict traditional impact indicators. Taken together, our findings indicate an interdisciplinary difference in the predictive value of Facebook likes, according to which Facebook likes only predict citations in the psychological area but not in the non-psychological area of business or in the field of life sciences. Our paper contributes to understanding the possibilities and limits of the use of social media indicators as potential early indicators of the impact of scientific work.

## Introduction

Assessing and evaluating the impact of research articles is a fundamental process in science that serves the advancement of knowledge in our society [1]. Finding relevant research, processing (current) research, and evaluating research are increasingly difficult and time-consuming undertakings [2,3]. Although performance assessments based on specific quantitative indicators (e.g., counting published journal articles) are fiercely criticized in the literature due to their neglect of qualitative aspects of scientific performance [1,4,5], the strong interest in performance evaluations to indicate the impact of scientific work is understandable [5–7].

Traditional indicators have severe disadvantages with respect to the assessment of scholar’s past and future performance [8] and the assessment of the impact of scientific articles. First, traditional indicators exhibit general shortcomings such as the distortion of the Impact Factor [9] and the fact that traditional indicators take months to years until they are able to indicate any influence on science. Time delays partially result from the publication delay, i.e., from the time delay between the preparation of a manuscript and its actual publication date (this time frame can encompass several years, e.g., when publishing in leading management journals) [10]. Time delays are also caused by the citation gap, i.e., the time delay between the publication date of a manuscript and the publication dates of subsequent manuscripts that cite the manuscript [11]. Second, the drastic increase in the amount of scientific work being produced [12] requires a different approach (metaknowledge) [3], i.e., faster processing and a clearer indication of relevant articles. Journal article citations may resemble a good retrospective indicator. However, citations accumulate rather slowly. Hence, they do not provide scholars and other stakeholders in science much assistance with daily decisions on the increasing volume of (fresh) work (e.g., to identify relevant articles to read and work with or to identify increasingly popular research topics) [2]. Furthermore, the impact of scientific work extends beyond the formal scientific discourse [13]. Thus, a suitable indicator for the impact of scientific work that reduces information overload is required to assist the stakeholders in science.

The aforementioned shortcomings of traditional indicators and the multitude of scientific performance dimensions [2,14,15] led to the development of innovative, alternative measures (altmetrics) [2,13]. Altmetrics is “the study and use of scholarly impact measures based on activity in online tools and environments” [2] and encompasses, for example, Tweets as well as downloads and views on open access or open review websites [16]. Despite the espoused importance of promptly assessing the impact of the increasingly growing (fresh) amount of scientific work, however, there is scant empirical research on early indicators for the impact of scientific work from the green open access road (i.e., scientific work not (yet) published in scientific journals [17]). Furthermore, the research has observed that social media content (Twitter and Facebook likes) can predict real-world attributes [18,19], such as personality traits, intelligence, and other personal attributes [19]. For instance, Google query volumes for search terms have been applied to detect real-world events, such as weekly transaction volumes [20] and stock market moves [21]. Stock market moves have also been shown to be predictable early on by the number of views of Wikipedia articles related to financial subjects [22] and by data from Google Trends [23]. Similarly, Twitter discussions have helped locate political demonstrations [24]. Thus, the Internet has become a crucial medium for gathering information [23]. In the area of science, prior work found that altmetrics such as Facebook wall posts, Tweets, and blog posts were related to journal article citations [16,25].

The study by Thelwall et al. [16] appears to be particularly noteworthy in this context. Based on a biomedicine and life sciences sample, Thelwall et al. [16] observed that six altmetrics (Tweets, Facebook wall posts, research highlights, blog mentions, mainstream media mentions, and forum posts) out of eleven altmetrics were strongly associated with citations of published articles in a simple sign test. Although this study offers a valuable contribution to the field of altmetrics research given that it contrasted eleven indicators, the study is limited because it “did not test that high altmetric scores today make high citations tomorrow more likely” [16]. Thus, conclusions with regard to the predictability of traditional impact measures by altmetrics cannot be drawn, and its value is unclear as an early indicator of scientific impact. In addition, the measure indicators do not represent a clear positive evaluation from the reader (i.e., Tweets can range from totally negative to totally positive in valence). This research gap is also valid for the extant literature that has not examined altmetric indicators of manuscripts that *advance* journal publication and traditional indicators. Another limitation of the study by Thelwall et al. [16], which is also valid for the remaining literature in this research field, is that it did not consider altmetrics that unambiguously *depict a positive attitude* of the reader (i.e., Facebook likes are only given for a manuscript if the reader has a clear positive attitude about the manuscript. In contrast, citations may also be inserted when one disagrees with, for example, the content/method of an article). Previous research is further limited because it seldom went *beyond journal article citations* by taking into account additional traditional impact indicators (e.g., Impact Factor) [26].

These oversights are noteworthy for the following reasons. First, early predictive indicators of the impact of scholars’ unpublished manuscripts are important, for example, for recruitment decisions on a practical level and the dynamic advancements of science. Early indicators could prevent (1) unknown parallel research agendas and (2) the delayed incorporation of important findings in current studies (note that much research is not (yet) available for all researchers because it is unpublished and thus cannot be taken into account by other researchers). Although one might argue that current parallel research agendas could generally be prevented by uploading manuscripts to the Internet, solely uploading manuscripts appears to be inefficient due to the massive amount of research that exists. Rather, the huge amount of new publications requires a filter [2] for the uploaded manuscripts to speed up the process of finding relevant manuscripts, authors or the like. Hence, there is an urgent need for a suitable early indicator of the impact of manuscripts.

Although such a filter cannot replace the critical reflection of scholars themselves, of course, it might be a helpful tool to find impactful research in one’s field more quickly and to build on the extant research in one’s own studies. Second, having early indicators with a clear positive semantic meaning appears to be inevitable to filter the vast amount of research. Only if an indicator has a clear positive semantic meaning can it be easily used in automatized ways to quickly filter articles. Third, scientific performance is multidimensional, and the impact on internal and external stakeholders in science cannot be equated [14]. Hence, using solely traditional indicators of scientific journal articles does not reflect this multidimensionality. Rather, the opinions and assessments of the impact of scientific work by external stakeholders (e.g., practitioners) and also by internal stakeholders (i.e., authors with similar research topics that are, however, not sufficiently related to actually cite a paper) have been largely neglected [14].

To address the aforementioned research gap, we analyze *Facebook likes* as a potential early indicator of the impact of unpublished scientific manuscripts that are uploaded on the Internet but not yet published in a journal. To date, social media features have not been widely used and accepted as alternative impact measures of scientific work. However, social media (e.g., Facebook) could provide valuable information to the multitude of scientific performance dimensions and could act as an information filter [27]. A startup realized the potential of Facebook likes in science and aims to use Facebook likes as an alternative to the Impact Factor to more rapidly assess the scientific reputation of manuscripts [28]; thus, Facebook likes also appear to be of utmost relevance in this context from a practical perspective. Investigating whether Facebook likes can forecast traditional offline indicators (e.g., journal article citations, Impact Factor, Immediacy Index) is important, on the one hand, because Facebook likes represent a potential impact measure that provides the relative impact information very fast and, on the other hand, because Facebook likes always express a clear positive semantic meaning (i.e., liking a manuscript indicates a positive influence).

We address the research gaps by (1) comparing traditional indicators of impact based on a thorough theoretical comparison and (2) using matched samples for which data on the Harvard Business School (HBS) and the bioRxiv websites, as well as from the Web of Science, were collected. The results partly support Facebook likes as an early indicator of the impact of scientific work.

The paper is structured as follows: First, we present the theoretical background. Second, we present our method and analyses of Studies 1 and 2. Finally, in the general discussion section, we note the theoretical and practical implications of our study and limitations and future research avenues.

## Theoretical Background

### Bibliometrics

Bibliometrics are used to evaluate scientific research output and to facilitate decision making for different stakeholders in science. Although the (current) (mis)use of bibliometrics is criticized in the literature [1,5,9,29,30], scholars may rely on bibliometrics to decide which studies are relevant and worth reading, or librarians might base their decision regarding which journals to subscribe to on bibliometrics. The scientific community can jointly act as a search engine [31] to reduce the information overload caused by the mass of articles, which a single person cannot read. There are many bibliometric indicators (e.g., citations, Impact Factor, Immediacy Index, h-index, h-core, e-index) that are supposed to indicate the scientific impact of scholarly work and scholars. Citations and the Impact Factor are of particular importance because they have been increasingly used in academia in the past several decades and because they often serve as a basis, for example, for evaluating past and for predicting future scholarly performance in tenure decisions or in grant decisions [1,6,8]. In the formulation by Penner et al. [7], “a tenure-track hire is a million dollar bet on a young scientist’s future success”, it becomes readily apparent why predictive indicators are sought after and critically evaluated.

Given the mass of scholarly work, a thorough investigation of indicators that are used to determine the impact of scientific articles (e.g., citations, Impact Factor, Immediacy Index) rather than an investigation of indicators that determine the impact of authors (e.g., h-index, h-core, e-index) appears to be particularly relevant. Therefore, in the following, we focus on citations, the Impact Factor, and the Immediacy Index (a variation of the Impact Factor), which are all easily accessible, for example, via the Web of Science website.

Citations are carefully selected posts left behind after the information of an article has been retrieved and used [32]. Thus, on the one hand, citations may indicate the relevance of an article and therefore serve as an indicator of an article’s popularity and impact [33,34]. On the other hand, using citations as a bibliometric indicator has a number of disadvantages. First, the valence or sentiment (i.e., the reason why an article is cited) can be positive or negative [15,31]. For example, an article could be cited to oppose the author’s arguments or to highlight methodological shortcomings, or an article could also be cited for non-scientific reasons [35]. Second, citations are distorted because the databases from which they are collected cover mainly English-language journal articles, and other types of literature are not (entirely) covered (e.g., national literature, books). Third, citations accumulate very slowly due to the publication delay [10] and due to the citation gap [11]. Finally, citations only display formal recognition of scholarly impact [2]. If an article is never cited, this indicates that *scholars* may not have referred to or used the article, but it does not mean that the article is useless to other stakeholders.

In addition to citations, the impact of journal articles is often estimated based on journal indicators (note that journal indicators were originally created for and used by librarians to compare journals within one discipline and not to compare single articles). A widely used indicator for journal quality and impact that is easy to understand and to interpret (also for non-academics), is the Impact Factor [36]. The Impact Factor is a mean value, and it is computed by “counting the number of current year citations to articles published by the journal during the preceding two years and dividing the count by the number of articles the journal published in those two years” [1].

However, there have been an increasing number of arguments against the Impact Factor [1,4,5,9]. First, the Impact Factor of a journal overstates the impact of many articles because it can be driven by a few highly cited articles [1], i.e., the Impact Factor is neither a good measure of the quality of journals, nor is it a good measure of the quality of a single article. The reason for this fact is that journals with a high Impact Factor can include many articles that receive few citations or no citations at all [1]. Second, despite annual updates, the Impact Factor is calculated based on the citations and journal publications of the preceding two years and thus is a retrospective measure with questionable validity to indicate the impact of scientific articles that have been accepted or published recently.

Another journal-level indicator is the Immediacy Index, which is calculated similarly to the Impact Factor; however, it is based on the articles published by the journal during the preceding year and not the preceding two years [37,38]. Due to the similarities in the calculation of the indicators, the Immediacy Index bears disadvantages comparable to those of the Impact Factor. Although the Immediacy Index differs from the Impact Factor by providing information about more recently published articles (greater weight to rapid changes), the Immediacy Index is also retrospective. However, due to the lower number of articles considered and, hence, likely fewer citations, the Immediacy Index might be influenced more easily by the impact of single publications.

The Impact Factor and the Immediacy Index have been investigated in the (bibliometric) literature as scientometric indicators. Research about the Impact Factor and Immediacy Index has, for example, found that the journal Impact Factor is a strong predictor for citations [39]. Furthermore, the information value and usefulness of the Impact Factor and Immediacy Index have been investigated and questioned [9,38,40] and compared to altmetrics [26].

Because academic performance is multidimensional [2,14], recently, scholars have started to differentiate between impact measures for internal and external stakeholders in science and have suggested using a variety of indicators. For example, Aguinis et al. [14] measured the impact of scholars via citations versus non-.edu pages and found that the rankings of scholars differed depending on the measure used. This multidimensionality and the vast amount of research that needs to be processed, as well as the publication delay [10] and citation gap [11], resulted in the call for a change in the evaluation of scientific output [29] and in the call for altmetrics (for a good overview of the characteristics of traditional and alternative indicators, see Torres et al. [41]). Currently, traditional and alternative indicators do not allow one to evaluate and to assess the impact of an article promptly. In other words, better filters (i.e., indicators) are needed to evaluate the vast amount of (fresh) research quickly and in a time-efficient manner. For this specific purpose, prior work suggested tools built on networks [15], as we live and work in increasingly complex and global digital networks [42–44]. The field of computational social science investigates individual and group behavior by using big data on human behavior offered by new information and communication technologies (ICT), for example, social media websites such as Facebook [42,43,45]. Computational social science research is exciting because it offers advantages in quantitatively analyzing collective behavior (e.g., due to the richness of the data) and can reveal differences in offline and online behavior [46,47]. Computational social science studies have, for example, investigated the effect of social influence on decision making about installing Facebook applications and identified a threshold of popularity for the applications, while the specific on-off nature of the social influence seems to be specific to the online environment [47]. The Facebook like button represents one example of such new technologies that appears to be worth investigating, for example, as a measure of the impact of scientific work. However, so far, studies in the computational social sciences on scientific work [48–50] only investigated, for example, worldwide citation flows, finding that citation flows decrease with geographical distance [51], and investigated the prediction of the impact of scholars [52]. The latter study indicated that future citations can be predicted on the basis of published articles (e.g., the h-index); however, future citations of future articles are difficult to predict. Another study in this vein reports that disciplinary fragmentation is limiting progress in science, while the driving factors are reported to be social interactions and peer disagreement [53]. However, studies on Facebook likes as an indicator to predict scientific impact are missing. In the next paragraph, we provide the reasons for which Facebook likes are a valuable possibility for assessing the impact of scientific work.

### Facebook Likes as an Altmetric

The literature has revealed that crowds can give valuable information, also known as collective wisdom [18,19]. Facebook users, being a crowd, already play a role as information filters [27]; for example, they use links on Facebook to search for and to share information with others. Interestingly, the higher the educational background of the users is [27], the more news content the users post on Facebook. Therefore, one might expect that scholars, being highly educated individuals, tend to use Facebook likes for sharing information as well, and thus, Facebook likes may be a valuable indicator of the impact of scholarly work. The existing literature has already revealed the antecedents of the Facebook liking process, such as the network degree [54]. Additionally, previous research has revealed that Facebook likes can predict, for example, personal attributes [19] or the probability of checking out music recommendations [55]. However, Facebook likes have not been investigated as a more contemporary early indicator of the impact of scientific manuscripts. Until now, altmetrics researchers have been more concerned with analyzing Tweets [16,25,56] rather than Facebook likes for assessing the impact of scientific work. Analyzing Facebook likes, however, appears to be very promising because, unlike Tweets and other previously analyzed altmetrics, Facebook likes convey the clearly positive attitude of the person liking a manuscript.

In addition to the fact that Facebook likes convey a clearly positive semantic meaning, Facebook likes offer a number of further advantages compared to traditional indicators. An advantage that appears to be of utmost importance is that Facebook likes could indicate the impact of a manuscript before it undergoes the time-consuming publication process. In addition, Facebook likes could provide direct feedback to authors regarding the rate and magnitude of the impact of their current work, in other words, whether their present work might become a raving success. Prior to the publication of an article in a journal, no Impact Factor or Immediacy Index is available. Facebook likes represent a form of open review for a broad scientific and non-scientific audience. Specifically, Facebook likes can recommend scientific manuscripts to a broad audience, even if this audience does not go on to cite the manuscript in a scientific article themselves (e.g., because the manuscript does not perfectly match the topic of their work or because they do not publish scientific articles and instead use the content of the manuscript for teaching or other practical work). Facebook likes thus allow for a broader inclusion of perspectives of various research stakeholders and additionally make the evaluation of science available to non-researchers.

Therefore, using Facebook likes as one potential indicator could better address the multidimensionality of scientific performance, as well as considering the impact on, for example, practice and society. Aside from a broader audience, the manuscripts uploaded on the Internet might also be broader and of a larger variety. Such manuscripts may cover non-mainstream research or studies with non-significant results that are generally more difficult to publish. The fact that a wider range of manuscripts will be available in a timely manner and that different stakeholders in science can contribute to filtering the increasing amount of scholarly work can facilitate the search for red-hot manuscripts. Additionally, Facebook likes may reduce self-interested referencing habits. Because it cannot be determined who liked a manuscript, there is no need to like a well-known author’s manuscript to flatter her/him. Furthermore, Facebook likes are independent from limited databases, such as Web of Science or Scopus. Unlike such databases, Facebook likes are not limited to specific journals, timeframes or work written in English. That is, contrary to such databases, Facebook likes do not neglect a great amount of scientific work, which is especially important for certain subjects such as legal sciences.

However, Facebook likes as an impact measure of scientific work also have disadvantages (see Table 1). Facebook likes can be easily manipulated; for example, authors can like their own work, thereby inflating the number of Facebook likes. Moreover, the informative value of Facebook likes for scientific manuscripts and the validity and reliability of Facebook likes are not clear. Facebook likes may be the result of a catchy title, an innovative scientific study or something else. In addition, Facebook likes might be more spontaneous, less thoughtful and less selective than citations. Finally, Facebook likes are not an established indicator in science and are scattered across websites. Nevertheless, the absence of a relationship of Facebook likes to traditional measures does not necessarily mean that Facebook likes are not a relevant impact measure; likes might rather address a different aspect of impact, which would be valuable to at least consider in the multidimensionality of scientific performance [14,57,58].

**Table 1:** Comparison of Facebook likes of a manuscript, journal article citations, and the Impact Factor/Immediacy Index as potential impact indicators of scientific work.

	Facebook likes	Citations	Impact Factor/Immediacy Index
Advantage	• Possibly alternative, more modern and faster index of the influence of an unpublished manuscript	• Citations are carefully selected posts after information of an article has been used	• Indicates the popularity of a journal in the scientific community in an easy to understand fashion
	• Possibly more direct feedback for authors (the rate and magnitude of the manuscript impact)	• Citations are strongly accepted and heavily consulted as an indicator of the impact of the quality and relevance of a journal article	• Impact Factor is a worldwide accepted standard indicator, e.g., for the comparison of journals and hiring decisions
	• May facilitate the search for red-hot manuscripts within the drastically increasing amount of scientific work		
	• Clearly renders a positive opinion of a manuscript in an open review form		
	• May include recommendations from stakeholders in science who read but may not cite the manuscript		
	• Manuscripts that might not be published in journals are also considered		
	• May reduce self-interested referencing habits (cannot be determined who liked a manuscript)		
	• Are independent from limited databases		
Disadvantage	• Unclear informative value (e.g., large number of likes may reflect social influence, catchy title)	• Unclear informative value (i.e., negative and positive citations cannot be distinguished)	• Invalid statements about single articles based on the skewed distribution of citations (and the number of published articles) in a journal in a given timespan
	• Can be manipulated (inflated)	• May be given for reasons other than appropriateness, e.g., to elevate citations of own articles	• Retrospective measure that is annually updated and does not necessarily reflect current publications
	• Might be given in a more spontaneous and less thoughtful way	• Are distorted: Limited databases that cover selected journals/timeframes/mostly work in English	
	• Is not an established indicator in science	• Long time lags until the influence of an article becomes apparent: publication delay, citation gap	
	• Likes are not centrally available and traceable for all manuscripts	• Display formal recognition of scholarly impact and do not necessarily depict to what extent the article was read or is use to non-scientists	

The Impact Factor and the Immediacy Index are discussed together in one column in this table because both rely on similar calculations and thus are associated with similar advantages and disadvantages.

Based on the reflections above, one might assume that the Facebook likes for uploaded manuscripts that have not been published in a journal predict traditional measures of impact (i.e., citations, Impact Factor and the Immediacy Index). One might argue that citations and Facebook likes are positively related to one another because both are scientific output indicators on the article level and both may assess some form of publicity and attention that a manuscript/article receives. One might also assume a positive relationship between Facebook likes and the Impact Factor, as well as the Immediacy Index, as research has revealed that article and journal measures can be related (e.g., citations and the Impact Factor, cf. Callaham et al. [39]). However, there are also suitable reasons to assume that there is no relationship between Facebook likes and the traditional impact measures considered in this article (i.e., citations, Impact Factor, Immediacy Index) because the stakeholders in science who use Facebook likes and thereby evaluate scientific work on the Internet versus those stakeholders in science who solely use traditional indicators might differ. That is, Facebook likes and traditional indicators may resemble different aspects of scientific performance observed from different perspectives. Our study, therefore, sets out to explore the relationship between Facebook likes and the specific traditional impact measures (i.e., citations, Impact Factor, Immediacy Index).

## Study 1

### Method

#### Sample and Procedure

For our matched sample, we first collected the number of Facebook likes for 300 manuscripts on the HBS Working Knowledge website (http://hbswk.hbs.edu/workingpapers). The HBS website provides the opportunity for advance online publishing of innovative manuscripts to enable the use of the manuscript’s content before it enters mainstream management practice. Information about the manuscript is provided on this website, and the manuscript is usually directly accessible via a link. We hereby confirm that we adhered to the terms of use for the HBS website and Facebook when conducting Study 1. Subsequent to the collection of the Facebook likes for the manuscripts, we checked whether the manuscripts that we identified on the HBS website were listed in the Web of Science database from Thomson Reuters. From the Web of Science, we collected the number of journal article citations, the Impact Factor of the journal in which the article is published, and the Immediacy Index. To determine whether the manuscript on the HBS website was the same as the journal article, we screened the author names, titles and abstracts of the manuscripts and journal articles. If the published journal article was exactly the same (*n* = 81) or the same with minor changes, for example, in terms of wording (*n* = 99), we included the respective manuscript in our analyses. If the manuscript uploaded on the HBS website was not found to be listed as such in the Web of Science database (*n* = 120), we dropped the manuscript from our sample prior to analysis.

Additionally, datasets with missing values in the variables under investigation were excluded (*n* = 9), for example, when the Immediacy Index was not available for a journal on the Web of Science. Finally, we deleted one manuscript from our dataset because the respective manuscript was published in a journal in the year prior to its upload on the HBS website, meaning that the number of Facebook likes on the HBS website cannot serve as a potential predictor of subsequent journal article citations (*n* = 1). This procedure left us with a final sample of *N* = 170 manuscripts. The data were collected in August 2013.

#### Measures

*Predictors*: We measured our predictor Facebook likes in two different ways. First, we collected the number of *Facebook likes* that were indicated by the Facebook Recommendation button for 300 scientific manuscripts uploaded between 2003 and 2010 on the HBS website. Second, we measured *non-zero Facebook likes* by excluding all manuscripts from the above mentioned 300 manuscripts that received zero Facebook likes (resulting in a sample of *n* = 95). The reason for introducing this second operationalization was that a high number of zero-values in Facebook likes might distort potential relationships. In addition, it is unclear what zero-likes precisely means (e.g., not liked by any reader, not read at all due to, e.g., no interesting title). Previous research in the field [16] has excluded non-zero-metric scores, too. Thus, rerunning our analyses with non-zero Facebook likes as a predictor appears to be appropriate.

*Control variable*: For each manuscript collected on the HBS website, we noted the year in which the manuscript was uploaded. Controlling for the *upload date* on the HBS website appears to be inevitable because manuscripts were uploaded in different years, and thus, the time to collect Facebook likes and citations varied between manuscripts. Controlling for time when analyzing altmetrics is also suggested in the literature [16,59].

*Criteria*: We collected bibliometric data from the Web of Science for the journal articles (published between 2004 and 2013) that were able to be matched to the collected manuscripts from the HBS website. In particular, we assessed the number of journal article *citations*, the two-year *Impact Factor* of the journals in which the articles were published and the journal *Immediacy Index*. The Impact Factor and the Immediacy Index were retrieved from the Journal Citation Reports Social Sciences Edition in the Web of Science.

### Results

#### Descriptive Results

Descriptive statistics and correlations are displayed in Table 2. Facebook likes did not correlate with citations (*r* = .07, *ns*), the Impact Factor (*r* = -.10, *ns*), or the Immediacy Index (*r* = -.03, *ns*). Similarly, non-zero Facebook likes did not correlate with citations (*r* = .13, *ns*), the Impact Factor (*r* = -.11, *ns*), or the Immediacy Index (*r* = -.05, *ns*).

**Table 2:** Descriptive statistics and correlations for Study 1.

	*Min*	*Max*	*M*	*SD*	(2)	(3)	(4)	(5)	(6)
(1) Facebook likes	0	237	4.46	18.94	1.00***	.12	.07	-.10	-.03
(2) Non-zero Facebook likes	1	237	7.98	24.83		.10	.13	-.11	-.05
(3) Upload date	2003	2010	2007.56	1.40			-.46***	-.06	.07
(4) Citations	0	84	13.82	17.66				.19*	.06
(5) Impact Factor	0.23	6.70	2.29	1.27					.67***
(6) Immediacy Index	0.04	2.03	0.49	0.41					

*N* = 170 (*n* = 95 for non-zero Facebook likes); *Min* = Minimum; *Max* = Maximum; *M* = Mean; *SD* = Standard Deviation.

Pearson’s correlations are displayed.

* *p* < .05;

** *p* < .01;

*** *p* < .001 (two-tailed).

#### Results of Regression Analyses

**Fig 1:**
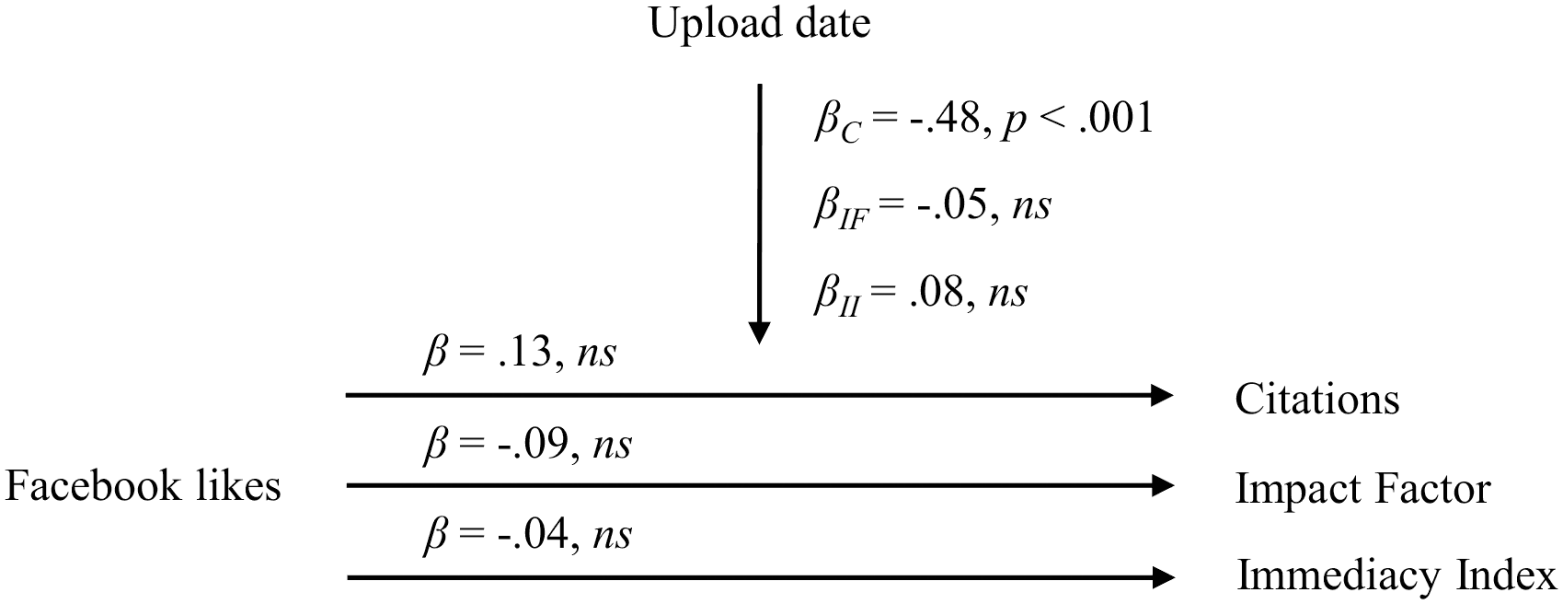
Relationship between Facebook likes of HBS manuscripts and citations, the Impact Factor and the Immediacy Index. *β*_*C*_ = Beta coefficient for the regression of the control variable upload date on the criterion citations; *β*_*IF*_ = Beta coefficient for the regression of the control variable upload date on the criterion Impact Factor; *β*_*II*_ = Beta coefficient for the regression of the control variable upload date on the criterion Immediacy Index.

Prior to our regression analyses, we z-standardized our variables. We first report the results for the predictor Facebook likes and then for the predictor non-zero Facebook likes. We observed that, when controlling for the upload date, the Facebook likes of manuscripts uploaded on the HBS website neither predicted citations (*β* = .13, *ns*), nor the Impact Factor (*β* = -.09, *ns*), nor the Immediacy Index (*β* = -.04, *ns*). The control variable upload date negatively predicted citations (*β* = -.48, *p* < .001); however, neither predicted the Impact Factor (*β* = -.05, *ns*) nor the Immediacy Index (*β* = .08, *ns*). An overview of the results of our regression analyses is presented in Fig 1.

**Fig 2:**
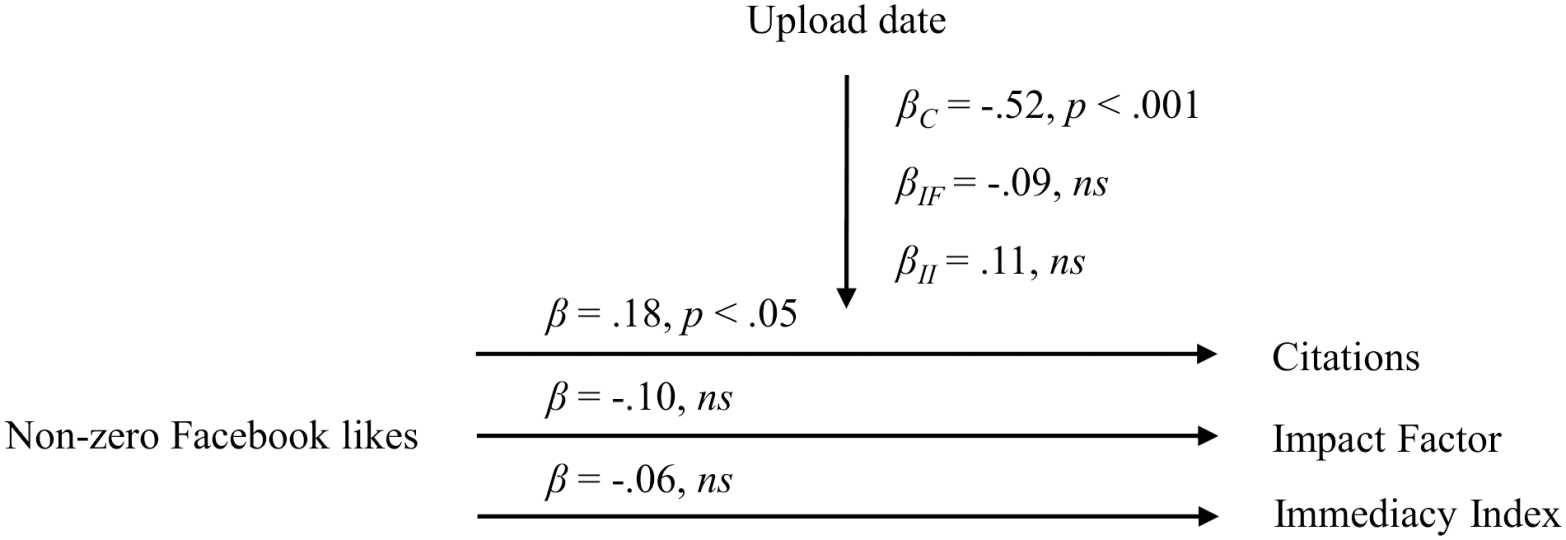
Relationship between non-zero Facebook likes for HBS manuscripts and citations, the Impact Factor and the Immediacy Index. *β*_*C*_ = Beta coefficient for the regression of the control variable upload date on the criterion citations; *β*_*IF*_ = Beta coefficient for the regression of the control variable upload date on the criterion Impact Factor; *β*_*II*_ = Beta coefficient for the regression of the control variable upload date on the criterion Immediacy Index.

Based on our regression analyses with non-zero Facebook likes as the predictor and controlling for the upload date, we observed that non-zero Facebook likes positively predicted journal article citations (*β* = .18, *p* < .05). However, non-zero Facebook likes neither predicted the Impact Factor of the journal the article was published in (*β* = -.10, *ns*) nor the Immediacy Index (*β* = -.06, *ns*). The control variable upload date negatively predicted journal article citations (*β* = -.52, *p* < .001), but this was not the case for either the Impact Factor (*β* = -.09, *ns*) or the Immediacy Index (*β* = .11, *ns*). An overview of our regression analyses results for non-zero Facebook likes is presented in Fig 2.

#### Additional Analyses

To test whether the results reported above can be generalized across scientific disciplines, we reran the regression analyses for two subsamples of the manuscripts from the HBS website that were included in our full sample (*N* = 170). In particular, two raters and one judge (who decided on the categorization in case of different categorizations between the raters) categorized the manuscripts into psychological (45.9%) and non-psychological manuscripts (54.1%).

For the psychological manuscripts, when controlling for the upload date, the regression analyses revealed that Facebook likes positively predicted citations (*β* = .21, *p* < .05), whereas they neither predicted the Impact Factor (*β* = -.07, *ns*) nor the Immediacy Index (*β* = -.02, *ns*). The control variable upload date negatively predicted citations (*β* = -.49, *p* < .001) but neither predicted the Impact Factor (*β* = -.14, *ns*) nor the Immediacy Index (*β* = .01, *ns*).

For non-psychological manuscripts, when controlling for the upload date, we observed that Facebook likes neither predicted citations (*β* = .05, *ns*) nor the Immediacy Index (*β* = -.13, *ns*); however, they negatively predicted the Impact Factor (*β* = -.26, *p* < .05). The control variable upload date negatively predicted citations (*β* = -.47, *p* < .001); however, it neither predicted the Impact Factor (*β* = .08, *ns*) nor the Immediacy Index (*β* = .14, *ns*).

Rerunning the regression analyses with the predictor non-zero Facebook likes, when controlling for the upload date, we found that non-zero Facebook likes for psychological manuscripts positively predicted citations (*β* = .24, *p* < .05), while they neither predicted the Impact Factor (*β* = -.09, *ns*) nor the Immediacy Index (*β* = -.05, *ns*). The control variable upload date negatively predicted citations (*β* = -.62, *p* < .001) but neither predicted the Impact Factor (*β* = -.22, *ns*) nor the Immediacy Index (*β* = .02, *ns*).

For non-psychological manuscripts, when controlling for the upload date, the regression analyses revealed that non-zero Facebook likes neither predicted citations (*β* = .15, *ns*), nor the Impact Factor (*β* = -.23, *ns*), nor the Immediacy Index (*β* = -.14, *ns*). The control variable upload date negatively predicted citations (*β* = -.39, *p* < .05); however, it neither predicted the Impact Factor (*β* = .15, *ns*) nor the Immediacy Index (*β* = .23, *ns*).

### Discussion

Based on the full sample, we found that Facebook likes of HBS manuscripts only predicted article citations when manuscripts with zero Facebook likes are excluded from the sample. The skewness of the Facebook likes (which is mainly because there are many manuscripts that received no Facebook likes at all) might be a potential explanation for the different findings based on the model using Facebook likes as the predictor versus using non-zero Facebook likes as the predictor. Zero Facebook likes could have different meanings: on one hand, zero Facebook likes might indicate that a manuscript was not approved of (and thus not liked) by readers; on the other hand, zero Facebook likes might indicate that a manuscript was never or seldom read, for example, due to the title selection or external reasons (e.g., when many manuscripts are uploaded at the same time). Thus, zero Facebook likes can have different semantic meanings and can result in a noisy measure. This unclear semantic meaning of zero Facebook likes might have undermined a relationship in our analyses including zero Facebook likes. Additionally, this unclear semantic meaning of zero Facebook likes emphasizes the importance of being critical about the meaning and use of zero Facebook likes.

Our results furthermore show that the number of (non-zero) Facebook likes a manuscript received was neither related to the Impact Factor nor the Immediacy Index. An explanation for this finding could be the fact that the traditional impact indicators are measured on different levels: Facebook likes (as well as citations) represent an article-level metric, while the Impact Factor and the Immediacy Index are journal-based metrics. There are studies that report a significant relationship between article and journal-level metrics [39]. Although these studies imply that different measurement levels, in general, can be mixed, our findings indicate that caution should be exercised with respect to the combination of journal-level metrics and article level-metrics, such as Facebook likes of manuscripts.

Additional analyses show that the predictive value of Facebook likes and non-zero Facebook likes only holds true for psychological manuscripts and not for non-psychological manuscripts on the HBS website. We suppose that there might be a difference in the acceptance of social media features, such as Facebook likes, across scientific disciplines (i.e., scholars working in different research fields might have different attitudes). However, it could also be the case that the readership of non-psychological manuscripts on the HBS website compared to non-psychological business journal articles differs, and their concept of influential scientific work varies, making predictions of citations by Facebook likes impossible. Accordingly, the results could indicate different stakeholders and the multitude of definitions of scientific performance.

Notably, in our additional analyses, we also observed that Facebook likes of non-psychological HBS manuscripts negatively predict the Impact Factor of the journal in which the manuscript is published. This result might be explained by the fact that what is liked on this more practical website (i.e., due to a more practical audience from the business domain) does not necessarily reflect what is published in influential business journals. A deviance between practice and science in business has been mentioned in the literature [34].

## Study 2

While additional analyses in Study 1 have shown that there are differences across disciplines in the predictive value of (non-zero) Facebook likes for citations, Study 1 is limited in its generalizability because the data were collected on one particular website. Therefore, in Study 2, we collected new data from a website in the life sciences.

### Method

#### Sample and Procedure

The data collection procedure corresponds to the procedure described in Study 1, except for the fact that we collected the data on the bioRxiv website (rather than on the HBS website). More precisely, we collected the number of Facebook likes of 600 manuscripts on bioRxiv (http://biorxiv.org/). On this website, unpublished manuscripts in the life sciences can be posted to make the study outcomes available to and receive feedback from the scientific community. Thus, bioRxiv represents a suitable website for Study 2 as it resembles the HBS website; however, it comprises manuscripts from another discipline, namely, the field of life sciences. On the bioRxiv pages, there are often links to the journal articles posted once the manuscript has been published in a journal. To determine whether the published journal article was identical (*n* = 145) or the same with minor changes (*n* = 189), we screened the author names, titles and abstracts of the manuscripts and journal articles. When no matching publication was found in the Web of Science, we dropped the manuscript from our dataset prior to the analyses (*n* = 266). Additionally, datasets with missing values in the variables under investigation were excluded (*n* = 62). Finally, prior to our analyses, we dropped two manuscripts from our sample because the respective manuscripts were published in a journal in the year prior to its upload on the bioRxiv website, meaning that the number of Facebook likes on the bioRxiv website cannot serve as a potential predictor of subsequent journal article citations (*n* = 2). We retained a final sample of *N* = 270 manuscripts. The data on bioRxiv and Web of Science were collected in April 2015. We hereby confirm that we adhered to the terms of use for Facebook and for the bioRxiv website when conducting Study 2.

#### Measures

*Predictors*: We collected the number of *Facebook likes* for 600 manuscripts uploaded on bioRxiv between 2013 and 2014. Furthermore, we measured *non-zero Facebook likes* by excluding all manuscripts that received zero Facebook likes, which resulted in a dataset of *n* = 84.

*Control variable*: The *upload date* is operationalized by the year in which the manuscript was uploaded on the bioRxiv website.

*Criteria*: We assessed the number of *citations*, the *Impact Factor* and the *Immediacy Index* for the journal articles as described in Study 1. The Impact Factor and the Immediacy Index were retrieved from the Journal Citation Reports Science Edition in the Web of Science. The assessed journal articles were published between 2013 and 2015.

### Results

#### Descriptive Results

Facebook likes neither correlated with citations (*r* = .06, *ns*), nor the Impact Factor (*r* = .08, *ns*), nor the Immediacy Index (*r* = .04, *ns*), as displayed in Table 3. Similarly, non-zero Facebook likes neither correlated with citations (*r* = .01, *ns*), nor the Impact Factor (*r* = .00, *ns*), nor the Immediacy Index (*r* = -.04, *ns*).

**Table 3:** Descriptive statistics and correlations for Study 2.

	*Min*	*Max*	*M*	*SD*	(2)	(3)	(4)	(5)	(6)
(1) Facebook likes	0	64	2.4	7.28	1.00***	.05	.06	.08	.04
(2) Non-zero Facebook likes	1	64	7.73	11.41		.10	.01	.00	-.04
(3) Upload date	2013	2014	2013.89	0.32			-.06	-.03	.00
(4) Citations	0	24	1.61	3.02				.36***	.34***
(5) Impact Factor	0.78	42.35	7.52	6.76					.95***
(6) Immediacy Index	0.04	12.04	1.51	1.52					

*N* = 270 (*n* = 84 for non-zero Facebook likes); *Min* = Minimum; *Max* = Maximum; *M* = Mean; *SD* = Standard Deviation.

Pearson’s correlations are displayed.

* *p* < .05;

** *p* < .01;

*** *p* < .001 (two-tailed).

#### Results of Regression Analyses

**Fig 3:**
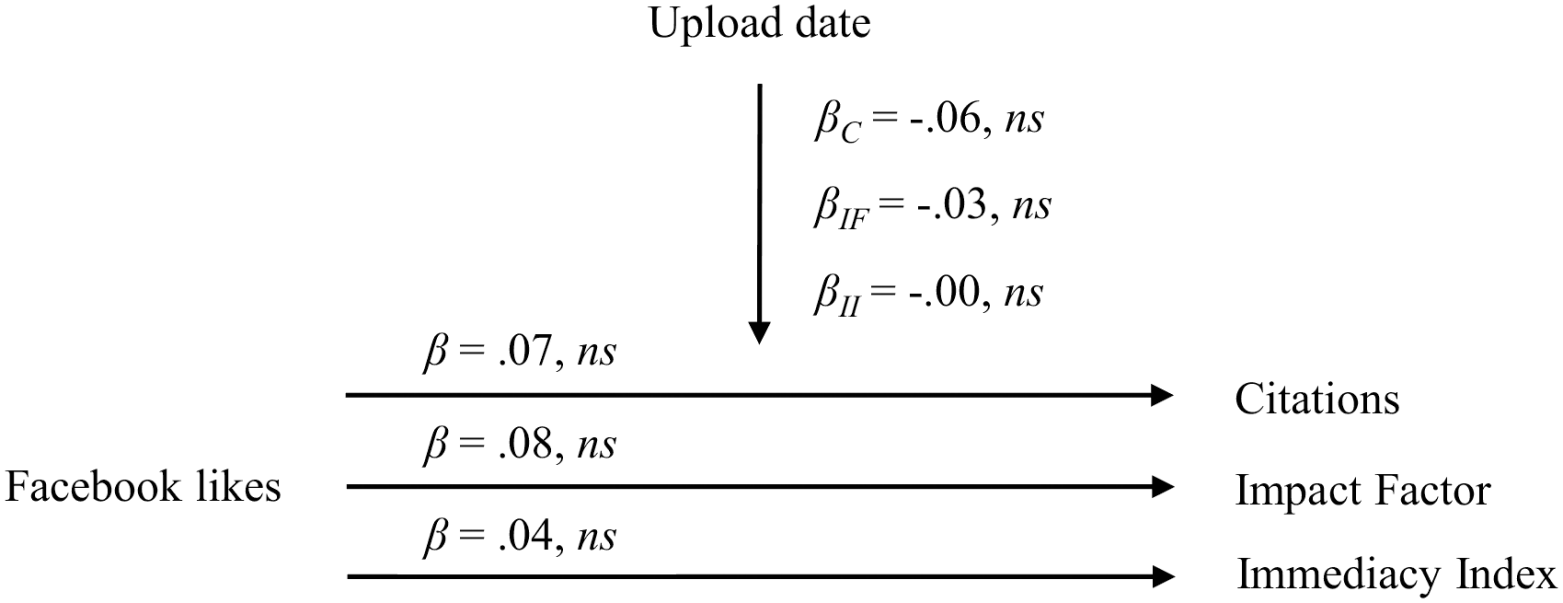
Relationship between Facebook likes of bioRxiv manuscripts and citations, the Impact Factor and the Immediacy Index. *β*_*C*_ = Beta coefficient for the regression of the control variable upload date on the criterion citations; *β*_*IF*_ = Beta coefficient for the regression of the control variable upload date on the criterion Impact Factor; *β*_*II*_ = Beta coefficient for the regression of the control variable upload date on the criterion Immediacy Index.

We z-standardized the variables prior to performing the regression analyses. When controlling for the upload date, Facebook likes of manuscripts uploaded on the bioRxiv website neither predicted citations (*β* = .07, *ns*), nor the Impact Factor (*β* = .08, *ns*), nor the Immediacy Index (*β* = .04, *ns*). The control variable upload date neither predictd citations (*β* = -.06, *ns*), nor the Impact Factor (*β* = -.03, *ns*) nor the Immediacy Index (*β* = -.00, *ns*). The results are presented in Fig 3.

**Fig 4:**
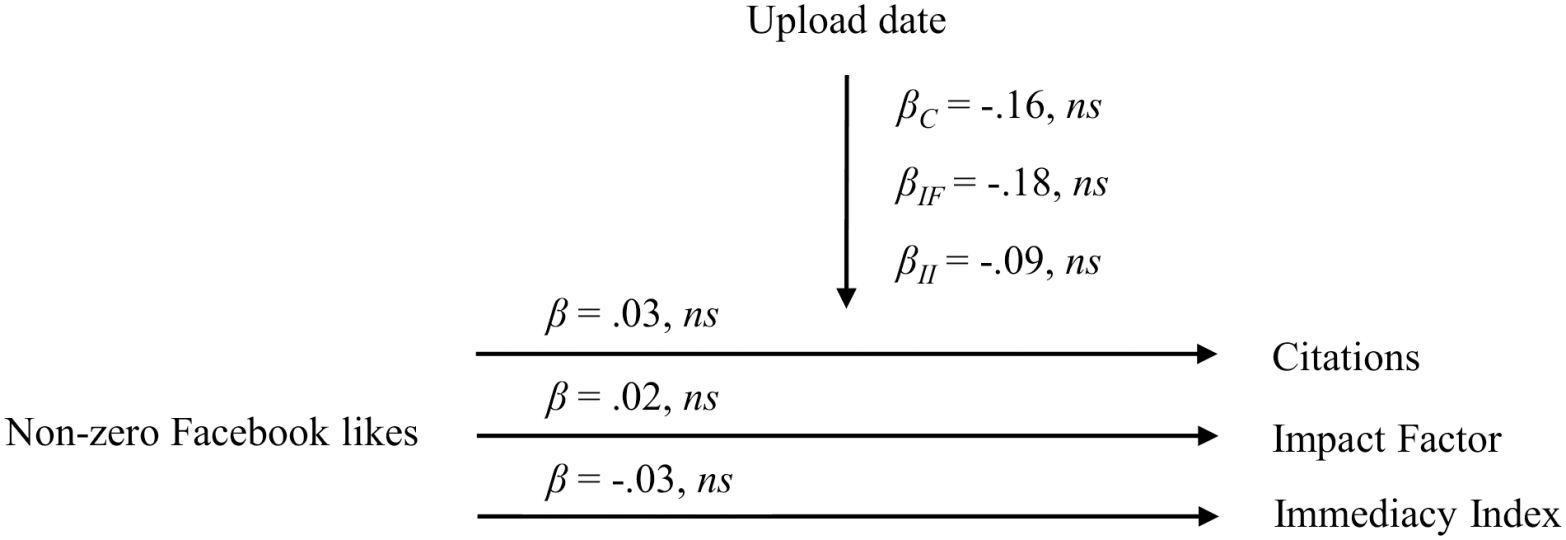
Relationship between non-zero Facebook likes of bioRxiv manuscripts and citations, the Impact Factor and the Immediacy Index. *β*_*C*_ = Beta coefficient for the regression of the control variable upload date on the criterion citations; *β*_*IF*_ = Beta coefficient for the regression of the control variable upload date on the criterion Impact Factor; *β*_*II*_ = Beta coefficient for the regression of the control variable upload date on the criterion Immediacy Index.

With non-zero Facebook likes as the predictor and controlling for the upload date, we find that non-zero Facebook likes neither predicted journal article citations (*β* = .03, *ns*), nor the Impact Factor (*β* = .02, *ns*), nor the Immediacy Index (*β* = -.03, *ns*). The control variable upload date neither predicted journal article citations (*β* = -.16, *ns*), nor the Impact Factor (*β* = -.18, *ns*), nor the Immediacy Index (*β* = -.09, *ns*). The results for non-zero Facebook likes are presented in Fig 4.

### Discussion

One explanation for the non-significant regression of non-zero Facebook likes on citations could be the relatively small sample size (*n* = 84). However, taking into account that the other regression analyses (*N* = 270) were also non-significant, the results indicate that (non-zero) Facebook likes of manuscripts uploaded on websites in the life sciences, such as bioRxiv, do not predict traditional impact indicators. One explanation for this finding could be that social media, such as Facebook likes, are not accepted for expressing whether one perceives a manuscript as influential. Another explanation is that the readership on the bioRxiv website versus the readership of life science journal articles differs and that their concept of influential scientific work differs, which makes predictions of citations impossible. Accordingly, the results may indicate different stakeholders and the multitude of definitions of scientific performance. A further explanation for the non-significant regression analyses on the Impact Factor and Immediacy Index may be that these indicators are journal-level metrics that do not relate to article-level metrics.

## General Discussion

This study set out to investigate Facebook likes as a potential early predictor of the impact of scientific work. Across both studies reported in this paper, we observed that Facebook likes of manuscripts cannot predict the journal-level indicators Immediacy Index or the Impact Factor, except for Facebook likes of non-psychological manuscripts from the HBS website, which negatively predict the Impact Factor. Another outcome of this paper is that citations are predicted by Facebook likes for psychological manuscripts from the HBS website. As indicated in our additional analyses, the significant prediction of citations by non-zero Facebook likes of manuscripts from the HBS website stems from the psychological and not from the non-psychological manuscripts on the HBS website. In Study 2, we observed no significant predictions of citations by Facebook likes of the manuscripts uploaded on bioRxiv. These outcomes demonstrate that Facebook likes may only have a predictive value in some disciplines. The data suggests that Facebook likes predict citations in the field of psychology but that they do not predict citations in, for example, life sciences. This interdisciplinary difference is also reflected in the fact that the HBS manuscripts (*N* = 170) that we categorized as being psychological in our study (45.9%) received 68.2% of the Facebook likes, whereas the manuscripts that we categorized as being non-psychological (54.1%) received 31.8% of the Facebook likes (note that similarly, 33.3% of the psychological manuscripts received no Facebook likes at all compared to 53.3% of the non-psychological manuscripts). Furthermore, interdisciplinary differences in social media behavior, i.e., uploading of papers [60] and the presence of altmetrics [59], have also been reported in the literature, with a higher involvement from social sciences and humanities [60]. Thus, these findings indicate a similar direction.

### Theoretical and Practical Implications

This paper contributes to the existing literature by comparing traditional impact indicators of scientific work with Facebook likes as an alternative impact indicator on a theoretical and empirical basis. Knowledge is extended on the usefulness of Facebook likes of unpublished manuscripts uploaded to the Internet to predict traditional indicators of impact. First, this paper reveals that the usefulness of altmetrics in the estimation of the (future) impact of an article depends on the discipline. In accordance with the literature, our findings demonstrate [60] that social media (Facebook likes) might be helpful in some disciplines as an additional measure (i.e., “addmetric”) rather than an alternative measure to traditional bibliometric indicators. While many stakeholders in science might prefer a few mutually agreed-upon impact indicators, this desire may not be accomplishable. Rather than replacing traditional impact measures, new measures should be regarded as additional metrics that might be able to enrich the impact assessment of scientific work [41,61]. As proposed in the literature, diverse metrics help to counter shortcomings of single indicators and the occurrence of a performance paradox [62]. Second, our findings suggest that journal impact measures are not useful as article-level metrics. This finding appears relevant because the Impact Factor is regularly used as an article-level metric in practice (e.g., in the evaluation of scholars in appointment or tenure decisions) [1,6].

Our findings also provide several practical implications. First, our findings suggest that Facebook likes can in some disciplines be a modern and prompt way of measuring and predicting the impact of a variety of (fresh) unpublished scientific work (that may not be covered in the Web of Science or other databases). Using Facebook likes of unpublished manuscripts reduces the problems of the publication delay and the citation gap. Hence, Facebook likes enable a rapid advancement of science and can better prevent parallel research on the same topic. Although indicators generally can also have disadvantages as exemplified by the performance paradox [62], Facebook likes can have benefits compared to other indicators (see Table 1). Second, and closely related, Facebook likes may in some disciplines represent an easy and at-a-glance indicator for the daily use of different stakeholders in science. Hence, one might think about, for instance, integrating the number of Facebook likes for Google Scholar search results. Additionally, further journal websites might implement the Facebook like button for each article, as Frontiers already does, and a German startup already aims to use Facebook likes to assess the scientific reputation of manuscripts [28]. Third, our theoretical considerations and empirical investigation suggest that Facebook likes may be beneficial as an addmetric because it provides an additional view on the manifold scientific performance. Following Neylon and Wu [15], we suggest collecting different article-level metrics for the assessment of the impact of scientific work. Because the impact of scientific work is complex, different stakeholders need different sources of information [15]. Stakeholders should base the decision on which specific indicators to use on the performance dimension(s) they are interested in. Hence, traditional and other alternative indicators might be useful as well.

### Limitations and Future Research

Our study is limited with regard to four aspects. First, Facebook likes represent an indicator that, as with other impact measures, can have shortcomings. According to the performance paradox, measuring performance based on specific indicators can lead to perverse learning and/or the neglect of unmeasured performance aspects [62,63]. Nonetheless, indicators also have their use in, for example, categorizing performance in a traceable manner. Furthermore, it is possible to manipulate Facebook likes; however, this behavior seems to be less relevant on websites, as Facebook likes currently do not represent a renowned indicator that scientists feel the need to excel in to advance their careers. Second, one might argue that the indicator Facebook likes is not suitable for assessing all facets of scientific performance from different stakeholder perspectives and that the scientific community may not consider Facebook a legitimate source of information that can be used to evaluate the impact of scientific work. While we acknowledge that these limitations might appear obvious, we feel confident that our implications are not limited in this respect for two reasons. First, based on the study by Baek et al. [27], we assume that scholars may use, for example, the Facebook like button just as non-scholars do because the authors report in their study that the frequency of posting links on Facebook is not related with the level of education. Baek et al. [27] interestingly found that the motives for posts differ. In particular, the study by Baek et al. [27] demonstrates that people with a higher education level post more links with news content than people with a lower education level. Thus, the purpose for using Facebook features rather than the frequency seems to vary for scholars and non-scholars. In another study, it was shown that 4.7% of scientific articles from a 2012 sample are shared on Facebook [60]. Considering that most publications usually receive zero or few citations [1], one might argue that a coverage of 4.7% of shared articles on Facebook [60] matches the usual scientific referencing pattern. Furthermore, differences with regard to the usage of social media have been found across scientific disciplines, with articles from the social sciences and humanities being represented to the largest degree [60]. Apart from social media use depending on education level and discipline, the information value that Facebook features have with respect to the impact of scientific work is of interest. One finding in the literature is that Facebook shares (in addition to, e.g., article views) can describe the early attention given to a publication [64]. Second, our confidence builds on empirical findings from a large international and interdisciplinary survey that we recently conducted. In our survey, we asked scholars, among other questions, whether at least one of their unpublished or published scientific manuscripts had been liked on one or more websites. Based on the full sample for this question (*N* = 913), we observed that almost one-third of our participants (29.9%) answered the question positively (i.e., checked “*yes*)”; 30.6% of our participants answered “*no*”, and 39.5% answered that the question was not applicable to them. Based on these findings, we argue that almost two-thirds of our respondents were obviously aware of the Facebook likes that their manuscripts received (or not) and that therefore, Facebook likes appear to be considered by scholars a legitimate source of information. Third, our study might be limited with respect to the predictive value of the collected Facebook likes. The collected Facebook likes might not have been given when the manuscript was just uploaded on the HBS website and could have been given anytime later, for example, after the publication of the manuscript in a journal. Thus, the predictive value of the Facebook likes might be reduced. Due to data constraints, it was not possible to control for the time when the Facebook likes were given. However, because research indicates that social media (Facebook shares and Tweets) has a quick uptake in the first few days after publication [64], it is suitable to assume that most Facebook likes were given in the first few days after the manuscript was uploaded or reported on the website. Fourth, this study investigates Facebook likes from two websites in the fields of (psychological versus non-psychological) business and life sciences. Thus, we cannot draw any conclusions with regard to other disciplines because publication practices and openness to social media might differ across disciplines. Fifth, the HBS website that we used to collect our data might be distorted as the manuscripts might all be of a comparatively high quality. The website is from a worldwide leading university with the majority of manuscripts written by Harvard scholars. In addition, HBS has a practical orientation. This practical orientation might be reflected in the type of manuscripts uploaded on the HBS website but also in the composition of people who like manuscripts on the website (i.e., readers). Furthermore, the website is marketed well and may, therefore, receive more attention than other websites of, for example, less famous universities around the world. These arguments might highlight the fact that the HBS website might be unrepresentative of other websites. However, as several articles in our sample have a rather low Impact Factor of, for example 0.23, there might also be variance in the quality of the manuscripts on the website. Thus, we are convinced that our findings contribute to extending our knowledge and that our study represents an important step toward the advancement of research on altmetrics (or addmetrics), especially because it provides valuable information on Facebook likes as an early predictor of the impact of scientific work. To address these issues, we also conducted Study 2 based on the bioRxiv website that seems to be less practically oriented than the HBS website and does not stem from such a world renowned institution such as Harvard.

Future research should address the following avenues of research. First, future research should investigate the reasons for differences between disciplines in the predictive value of Facebook likes, in other words, whether the interdisciplinary differences in the usage of Facebook likes arise from, for example, a differing acceptance of social media features or whether the differences emanate from a different readership of the uploaded manuscripts than those who read and cite scientific journal articles. Thus, it would be important as a first step to analyze those who give likes and then survey these people in terms of their attitudes toward social media. Second, tracking the exact date when a Facebook like was given to a manuscript would provide useful knowledge for the investigation of Facebook likes as an early indicator of the success and impact of one’s scientific work. Such tracking would enable researchers, for example, to control for whether a Facebook like has been given prior to or after the journal publication. Third, it would be interesting to investigate to what degree editors and journal reviewers, as well as scientists in general, (consciously) track Facebook likes for manuscripts and whether they are influenced by the Facebook likes of a manuscript with regard to quality ascription and, for example, the acceptance decisions of reviewers prior to the publication of a manuscript in a journal. In other words, does social media have an impact on the decision makers in science? A study that conducted similar research is the recently published work by Schulz and Nicolai [34]. In their study, they assessed the influence of the Harvard Business Review magazine and analyzed citations of the Harvard Business Review magazine in top-tier scientific journals; however, they did not assess the Facebook likes of manuscripts uploaded to a website and their influence on, for example, the decisions of editors. Fourth, a future avenue of research should examine in depth the information value of different traditional and alternative scientific performance metrics. In other words, the (here) investigated different indicators may cover different aspects of scientific performance, which may be observed from the perspective of different stakeholders and, hence, may only in part overlap. To give an example, it could be the case that Facebook likes may indicate something about the manuscript’s timeliness and practical relevance, while traditional indicators (i.e., citations) may indicate something about the article’s scientific novelty. In addition, the motivation for liking a manuscript may differ among the scientists and stakeholders in science. It would also be crucial to assess whether zero-Facebook like manuscripts result from not liking the manuscript or from other factors, such as few readers. Fifth, new research paths should be undertaken to evaluate the usefulness of alternative or additional indicators. For example different stakeholders, such as journalists and scientists, could evaluate scientific work in a blind-review procedure, and these qualitative evaluations could then be compared to traditional and alternative/additional impact indicators of scientific work. It might also be a worthwhile idea to assess the number of citations in the papers of a journal rather than solely checking whether an article is cited at least once, thus prompting it to appear in the reference list when research is conducted regarding citations. The number of citations in papers might better reflect how valuable and influential a cited article is than whether it appeared in the reference list. Sixth, we suggest comparing other external impact measures of scholarly work with Facebook likes, for instance, Google entries on non-academic servers [14], to address the multitude of aspects of scientific performance. Finally, we suggest further investigating altmetrics and addmetrics, such as post-publication reviews, comments and blog posts on scholarly work, for example, the PLOS ONE, Frontiers and F1000 websites; posting a comment may, for example, require more reflection and a more thorough reading of the manuscript than liking a manuscript. In studies that analyze comments, the frequency of comments (as with the frequency of citations) cannot necessarily be equated with the extent of positive evaluation by the community, as comments might critique the scientific work. Studies on blog posts on the ResearchBlogging.org website reveal the potential of (early) blog citations to predict citations [13,65]. In addition to investigating further measures, it might also be fruitful to evaluate other research publication procedures, which may resolve current shortcomings in the publication process. For example, “online first” publication processes, which can shorten the publication delay tremendously, can make research accessible earlier and speed up the scientific process.

## Conclusion

In summary, our paper contributes to the assessment of the impact of scientific work via social media indicators. We compared Facebook likes for manuscripts with traditional indicators of success and find that Facebook likes may be used as an addmetric and early impact indicator of psychological manuscripts, not, however, manuscripts from non-psychological areas of business or the area of life sciences. In light of the discipline-depending results, Facebook likes at least in part resemble something other than the impact measured by traditional impact measures. Thus, we are convinced that science would benefit from an open-minded allowance of diverse indicators to assess the multitude of aspects of scientific performance observed from the angle of different stakeholders. However, applying diverse indicators and assessment methods should be performed in a reflective manner, keeping the shortcomings of these indicators in mind. The contributions of our paper are relevant for scholars themselves and for the governance of academia, (social) media, society and other stakeholders. Future research in this direction is desirable to clarify the specific type of impact that Facebook likes measure and also who likes manuscripts in different disciplines. Our study is also conducive to elucidating the validity and reliability of Facebook likes as an addmetric.
